# Minimizing Breast Implant Contamination in Breast Reconstruction Procedures: Introducing the “12 Breast Reconstruction Points”

**DOI:** 10.1097/GOX.0000000000002028

**Published:** 2018-12-14

**Authors:** Barbara Cagli, Rosa Salzillo, Adriano Santorelli, Stefania Tenna, Paolo Persichetti

**Affiliations:** From the * Department of Plastic, Reconstructive and Aesthetic Surgery, “Campus Bio-Medico” University of Rome, Italy; † Plastic Surgery, Clinica A. Grimaldi, S. Giorgio a Cremano, Naples, Italy.

Reading with great interest the articles by Deva et al.,^1^ Adams et al.,^2^ and Gowda et al.,^3^ we noticed that little evidence is present about the application of the 14-point plan in implant-based breast reconstruction.

From January 2017, we applied and adapted the 14 points on 165 patients in every breast reconstruction procedure, to maximize patient safety and minimize complications’ rate:

1. Use intravenous antibiotic prophylaxis at the time of anesthetic induction: 2 g of cefazolin.
2. Avoid periareolar incisions: we use the “S”-shaped incision in the upper external quadrant or the inframammary fold incision for both demolitive and reconstructive purposes.
3. Use nipple shields: when nipple sparing mastectomy is performed we use nipple shields to prevent spillage of bacteria into the pocket.
4. Perform careful atraumatic dissection: the fourth point is not applicable in breast reconstruction because mastectomy is a highly invasive traumatic dissection that can lead to de-vascularized tissue.
5. Perform careful hemostasis.
6. Avoid dissection into the breast parenchyma: The breast parenchyma is completely removed during mastectomy. After the demolitive procedure, the pocket is washed with saline solution, new instruments and drapes are used.
7. Use a dual plane pocket: We place the implant in a total submuscular pocket or in a “modified” dual-plane pocket, with the inferior third of the implant being covered by subcutaneous tissue of the mastectomy flap or by a mesh/acellular dermal matrix.
8. Perform pocket irrigation with correct proven triple antibiotic solution or betadine: we wash the pocket and the implant, injecting the solution in the closed box in a sterile manner, with cefazolin 1 g and gentamicin 80 mg.
9. Minimize skin implant contamination: we don’t use introduction sleeves, but we clean the skin with betadine and apply a new steri-drape around the incision.
10. Minimize the time of implant opening and replacement of implant or sizer: the implant is opened when introduced and never replaced.
11. Change surgical gloves before handling implant and use new or cleaned instruments.
12. Avoid use of a drainage tube where possible: due to the combined demolitive and reconstructive procedures, patients present a considerable amount of fluids that require the use of drainage tubes to avoid postoperative seroma.
13. Use a layered suture.
14. Use antibiotic prophylaxis to cover subsequent procedures that breach the skin or mucosa.

In most cases, we found possible the application of 12 points out of the 14 points suggested by Deva et al.^1^

When the nipple-areolar complex is removed or the implant is totally submuscular, we don’t apply the third and the seventh point, making possible the application of 12 points out of 14. To our knowledge, this is the first report investigating the effectiveness and feasibility of the 14 point-plan on a population of breast reconstructive patients only.

As of this date we are not able to draw any conclusions because the follow-up is still short, but these preliminary data encourage us to continue following the “12 breast reconstruction points.”
